# Self-Collection for Primary HPV Testing: Perspectives on Implementation From Federally Qualified Health Centers

**DOI:** 10.5888/pcd20.230056

**Published:** 2023-10-19

**Authors:** Amanda Le, Catherine Rohweder, Stephanie B. Wheeler, Jennifer Elston Lafata, Randall Teal, Kara Giannone, MaryShell Zaffino, Jennifer S. Smith

**Affiliations:** 1 Department of Public Health Leadership, Gillings School of Global Public Health, University of North Carolina at Chapel Hill; 2 Center for Health Promotion and Disease Prevention, University of North Carolina at Chapel Hill; 3 Department of Health Policy and Management, Gillings School of Global Public Health, University of North Carolina at Chapel Hill; 4 Lineberger Comprehensive Cancer Center, University of North Carolina at Chapel Hill; 5 Division of Pharmaceutical Outcomes and Policy, Eshelman School of Pharmacy, University of North Carolina at Chapel Hill; 6 Connected Health Applications and Interventions, University of North Carolina at Chapel Hill; 7 Blue Ridge Health, Hendersonville, North Carolina; 8 Department of Epidemiology, Gillings School of Global Public Health, University of North Carolina at Chapel Hill

## Abstract

**Introduction:**

Primary testing for high-risk human papillomavirus (HPV) by self-collection could result in higher rates of cervical cancer screening. Federally qualified health centers (FQHCs) in the US serve a large proportion of women who have low income and no health insurance and are medically underserved — risk factors for being insufficiently screened for cervical cancer. Although the implementation of self-collection for HPV testing is not yet widespread, health care entities need to prepare for its eventual approval by the US Food and Drug Administration. We conducted focus groups and interviews among clinical and administrative staff and leadership to gather data on key logistical concerns that must be addressed before implementing self-collection for HPV testing in FQHCs.

**Methods:**

We identified focus group and interview participants from 6 FQHCs in North Carolina. We conducted focus groups with clinical and administrative staff (N = 45) and semistructured interviews with chief executive officers, senior-level administrators, chief medical officers, and clinical data managers (N = 24). Transcripts were coded by using codebooks derived from research questions and notes taken during data collection. Themes emerged on implementation of self-collection for HPV testing. We applied the constructs from the Consolidated Framework for Implementation Research (CFIR) to themes to identify domains of potential barriers and facilitators to implementation.

**Results:**

Clinical personnel reported that offering self-collection for HPV testing is acceptable and feasible and can increase cervical cancer screening rates. Uncertainties emerged about accuracy of results, workflow disruptions, financial implications, and effects on clinic quality measures.

**Conclusion:**

Implementing self-collection for HPV testing was considered feasible and acceptable by participants. However, important health service delivery considerations, including financial implications, must be addressed before integrating self-collection for HPV testing into the standard of care.

**Summary**

**What is already known on this topic?**

Most cervical cancer cases in the US are attributable to inadequate screening. Primary testing for high-risk human papillomavirus (HPV) by self-collection could result in higher rates of cervical cancer screening. Although most studies of self-collection for HPV testing focus on patient acceptability, little information exists on health service delivery considerations for implementing self-collection for HPV testing into clinical care.

**What is added by this report?**

We conducted focus groups and interviews with frontline clinical staff and leadership to gather data on key logistical concerns that must be addressed before implementing self-collection for HPV in federally qualified health centers.

**What are the implications for public health practice?**

Self-collection for HPV testing has the potential to increase cervical cancer screening rates among women overdue for screening.

**MEDSCAPE CME**

In support of improving patient care, this activity has been planned and implemented by Medscape, LLC and *Preventing Chronic Disease*. Medscape, LLC is jointly accredited with commendation by the Accreditation Council for Continuing Medical Education (ACCME), the Accreditation Council for Pharmacy Education (ACPE), and the American Nurses Credentialing Center (ANCC), to provide continuing education for the healthcare team.

Medscape, LLC designates this Journal-based CME activity for a maximum of 1.0 AMA PRA Category 1 Credit(s)™. Physicians should claim only the credit commensurate with the extent of their participation in the activity.

Successful completion of this CME activity, which includes participation in the evaluation component, enables the participant to earn up to 1.0 MOC points in the American Board of Internal Medicine’s (ABIM) Maintenance of Certification (MOC) program. Participants will earn MOC points equivalent to the amount of CME credits claimed for the activity. It is the CME activity provider’s responsibility to submit participant completion information to ACCME for the purpose of granting ABIM MOC credit.

Release date: October 19, 2023; Expiration date: October 19, 2024

**Learning Objectives**

Upon completion of this activity, participants will be able to:

1. Assess the characteristics of human papillomavirus self-collection kits
2. Evaluate the potential benefits of human papillomavirus self-collection from the perspective of federally qualified health center clinical staff and leadership
3. Evaluate the potential liabilities of human papillomavirus self-collection from the perspective of federally qualified health center clinical staff and leadership
4. Distinguish the preferred means of mode of delivery for human papillomavirus self-collection kits

**Credit Hours — 1.0**

EDITOR

Ellen Taratus, MS, ELS

AUTHORS

Amanda Le, MPH, Department of Public Health Leadership, Gillings School of Global Public Health, University of North Carolina, Chapel Hill, North Carolina

Catherine Rohweder, PhD, MPH, Center for Health Promotion & Disease Prevention, University of North Carolina, Chapel Hill, North Carolina

Stephanie Wheeler, PhD, MPH, Center for Health Promotion & Disease Prevention, University of North Carolina, Lineberger Comprehensive Cancer Center, University of North Carolina, Chapel Hill, North Carolina

Jennifer Lafata, PhD, Lineberger Comprehensive Cancer Center, University of North Carolina, Division of Pharmaceutical Outcomes and Policy, Eshelman School of Pharmacy, University of North Carolina, Chapel Hill, North Carolina

Randall Teal, MA, Lineberger Comprehensive Cancer Center, University of North Carolina, Connected Health Applications and Interventions (CHAI-Core), University of North Carolina, Chapel Hill, North Carolina

Kara Giannone, MPH, Lineberger Comprehensive Cancer Center, University of North Carolina, Connected Health Applications and Interventions (CHAI-Core), University of North Carolina, Chapel Hill, North Carolina

MaryShell Zaffino, MD, Blue Ridge Health, North Carolina

Jennifer Smith, PhD, MPH, Lineberger Comprehensive Cancer Center, University of North Carolina, Department of Epidemiology, Gillings School of Global Public Health, University of North Carolina, Chapel Hill, North Carolina

CME AUTHOR

Charles P. Vega, MD, Health Sciences Clinical Professor of Family Medicine, University of California, Irvine School of Medicine

Charles P. Vega, MD, has the following relevant financial relationships: Consultant or advisor for: Boehringer Ingelheim Pharmaceuticals, Inc.; GlaxoSmithKline; Johnson & Johnson

## Introduction

The landscape of cervical cancer screening has changed considerably in recent years. The US Preventive Services Task Force recommends testing for high-risk human papillomavirus (HPV) through use of health care provider–collected cervical samples for primary screening among people aged 30 years or older (1), alone or with cytology (co-testing). For people aged 21 to 29 years, cytology alone is recommended (1,2).

Most cervical cancer cases are attributable to inadequate screening (3). Because of systemic barriers, women without health insurance, in racial and ethnic minority groups, and living in rural areas are more likely than their counterparts to be overdue for screening, and thus, have a higher risk for cervical cancer (4). Federally qualified health centers (FQHCs) often operate over capacity and serve a large proportion of underscreened women (5).

Self-collection for HPV testing can improve screening rates by allowing women to collect cervicovaginal samples themselves. Self-collection is as sensitive as provider collection for HPV testing for detecting high-grade cervical precancers and cancers, if highly sensitive assays are used (6,7). Offering primary screening by self-collection to women overdue for clinic-based screening has resulted in higher rates of HPV screening than routine opportunistic screening (8,9). Although most studies of self-collection for HPV testing focus on patient acceptability (9–13), little information exists on health service delivery considerations for implementing self-collection in clinical care.

We aimed to understand determinants for a successful integration of an intervention for self-collection for HPV testing into service delivery at FQHCs. We interviewed FQHC leadership and clinical and administrative staff to identify barriers and facilitators for implementation, motivation, and willingness to implement self-collection, and logistical, organizational, and health service resource needs. These data are essential to obtain now, before the possible approval of self-collection for HPV testing by the US Food and Drug Administration (FDA). Our focus on a self-collection intervention to improve HPV screening uptake among underscreened women will inform clinical and policy interventions to pave the way for more rapid and widespread uptake of this evidence-based intervention.

## Methods

### Study setting and design

**Clinic identification.** In collaboration with the North Carolina Community Health Center Association, we selected 6 of 40 eligible FQHCs in North Carolina for participation, and all agreed to participate. We systematically selected the 6 FQHCs on the basis of their size and geographic location to ensure representation of FQHCs of various sizes in both urban and rural catchment areas. Two small FQHCs (consisting of 1 or 2 clinical sites) served urban and rural areas; 2 medium FQHCs (consisting of 3 to 6 clinical sites) served urban and rural areas; and 2 large FQHCs (consisting of >6 clinical sites) served rural areas. We used zip codes to determine rural and urban classifications. A representative of each FQHC helped recruit participants for focus groups and semistructured interviews.

**Focus groups.** We conducted 6 focus groups; 6 to 10 participants from each FQHC participated in each focus group. Health care provider and staff roles consisted of physicians (n = 7), physician assistants (n = 3), medical assistants (n = 3), nurses (n = 16), nursing assistants (n = 1), and administrative staff (n = 15), for a total of 45 study participants. Small FQHCs had 19 participants across 2 focus groups, medium FQHCs had 13 participants across 2 focus groups, and large FQHCs had 13 participants across 2 focus groups. Focus groups facilitated idea brainstorming, information gathering on organizational culture and norms, and uncovering factors that influence opinions, behaviors, or motivations for the implementation of self-collection for HPV testing. Focus group discussions were intended to increase understanding of the perceptions among FQHC clinical and administrative staff on introducing and implementing HPV self-collection among women overdue for cervical cancer screening. Each clinic was compensated up to $500 for participation.

**Semistructured interviews.** We selected 24 interview participants from the 6 FQHCs on the basis of job title and professional role: chief executive officer (CEO) (n = 6), chief medical officer (CMO) (n = 6), senior-level administrator (n = 6), and clinical data manager (n = 6). One participant in each role was selected from each of the 6 FQHCs. Interviews provided detailed information about staff roles in implementing an intervention for self-collection for HPV testing and expert opinions about the benefits of, or concerns about, its use. Key implementation issues that emerged from focus group discussions were used to develop interview guides and conduct follow-up semistructured one-on-one interviews to better understand key clinic personnel and administrative decision-making perceptions of the impact of implementation on clinics. Each participant received a gift card with a value up to $100  for completing the interview.

**Focus group and interview guides.** The Consolidated Framework for Implementation Research (CFIR) helped the study team to conceptualize themes for the focus group and interview guides (14). The CFIR considers factors related to implementation in 5 major domains: 1) inner setting (What potential facilitators and barriers would affect willingness to implement an intervention for self-collection for HPV testing?), 2) individual characteristics (What are the perceived benefits to and potential areas of pushback against adopting self-collection? Do FQHC administrators and staff believe self-collection is an appropriate intervention for their patients’ needs?), 3) outer setting (What external pressures, performance metrics, or other considerations encourage or discourage efforts to improve cervical cancer coverage among their patients?), 4) intervention characteristics (What resources are needed to implement an intervention for self-collection?) and 5) implementation process (How would staffing, scope of practice, and workflows need to change?).

### Data collection procedures

**Focus group procedures.** We conducted focus groups from January 2020 through March 2021. Facilitators followed structured focus group guides to elicit insights from FQHC staff. The first 2 focus groups were conducted in person. Because of the COVID-19 pandemic, the remaining 4 focus groups were conducted virtually through a cloud-based video conferencing platform that enabled audio recording. Study background material was provided, and informed consent was obtained before discussions, which lasted approximately 60 minutes.

**Semistructured interview procedures.** Semistructured interviews were conducted from May through September 2021. After informed consent was obtained, interviews were completed via telephone, lasting approximately 30 to 45 minutes. Interviewers followed 2 interactive, participant-focused interview guides driven by results from the focus group discussions: one was used for interviews with CEOs, CMOs, and other senior-level administrators, and another was used to guide interviews with clinical data managers. We excluded data obtained from interviews with clinical data mangers because those discussions provided an understanding only of electronic health record (EHR) systems and their capabilities in place at their respective health centers.

The University of North Carolina Institutional Review Board reviewed and approved the study protocol (no. 19–1639). Our study qualified as exempt from human studies research given that no sensitive information was obtained, and it involved only minimal risk to participants. Informed consent was obtained from all participants included in the study.

### Data analysis

All focus group discussions and interviews were digitally recorded. Files were transcribed and transcripts were imported into Dedoose version 9.0.17 (Sociocultural Research Consultants, LLC), a qualitative research software management tool, to facilitate analysis. We developed codebooks based on the research questions and notes taken during data collection. For focus group transcripts, an initial codebook was pilot tested by independently coding a randomly selected focus group transcript. We pilot-tested 2 additional codebooks (one for CEOs, CMOs, or senior-level administrators and one for clinical data managers) by independently coding several interview transcripts. A consensus coding approach was used, where codebooks were developed, piloted, and reconciled, leading to refinements to an updated codebook until replication was achieved across coders (15–17). Final codes were then applied to the remaining focus group transcripts by 2 independent coders and to the interview transcripts by 4 independent coders, for a total of 6 coders. We generated code reports for each code. Narrative summaries were written with descriptions of the themes and subthemes that emerged, and illustrative quotes highlighted each theme. We then organized themes into CFIR domains.

## Results

### Focus groups

Focus group participants described several benefits of self-collection for HPV testing, which were organized into the following CFIR domains: individual characteristics, intervention characteristics/implementation process, and inner setting (Table 1).

**Table 1:** Benefits of Implementing Self-Collection for HPV Testing in Federally Qualified Health Centers: Results of Focus Groups (N = 6) Conducted Among Clinical Personnel (N = 45), North Carolina, January 2020–March 2021

CFIR construct^a^	Theme	Illustrative quotes on benefits of HPV self-collection
Individual characteristics	Increase screening rates and care reach	[S]elf-testing theoretically will increase the amount of people that you screen. . . . . So, if you allow people to self-swab . . . they are more likely to want to do it even if they do it onsite. They may go in the bathroom and swab and bring it out. (Focus group 1 [small, rural])
	Acceptability	• I would like the idea only if the rollout of the test ensures that patients have the information to perform the self-collection, demonstration . . . [with] equity and health literacy around the entire process . . . especially for the populations of patients we serve. I believe in expanding access and meeting patients where they are, and if that does that and increases screening, that’s excellent, but if it’s just checking a box, no way. (Focus group 6 [medium, rural]) • [I]t can definitely increase access to care. . . . [S]pecimen swabs can be mailed to the patient’s home, they can do a self-collection . . . then mail those specimens back out to the laboratory facility, or the health center, who would then process them. (Focus group 3 [large, rural])
	Feasibility	[I]t’s a good idea comparing it to . . . our colorectal cancer screening. . . . [O]nce we started offering free testing, they didn’t have to come in for an appointment for it. They could . . . pick up the FIT [fecal immunochemical test] test and . . . bring it back . . . later, my numbers improved significantly. (Focus group 4 [medium, urban])
Intervention characteristics/implementation process	Kit distribution in clinic	The kit at the clinic when they come in … for any reason. They’re able to do it in the clinic. It’ll improve our numbers. (Focus group 4 [medium, urban])
	Kit distribution via mail	[We can] look through our registry and determine who would be eligible for that and mail those out or call them and let them know we’ve mailed it. (Focus group 5 [large, rural])
	Kit distribution via community outreach events	Send providers out to the community. . . . I think that the clinic would definitely allow time if that was something that we showed interest in. (Focus group 2 [small, urban])
	Obtaining results for self-collected tests	[B]ut if it’s a company that’s directly distributing the kits, they’re likely to be sending us reports rather than where we’re referring out. . . . So, it may actually be easier to track. (Focus group 1 [small, rural])
Inner setting	Timesaver	• [I]t could be a . . . provider timesaver per visit. (Focus group 2 [small, urban]) • I think the value would lie in freeing up clinic appointments and time. . . .[I]f you have a negative HPV, that’s a time that we could move on not having to do the Pap. So, I think that’s the value . . . and knowing we’ve . . . screened. (Focus group 5 [large, rural])
	Financial impact/billing	[If] we wouldn’t be doing the billing for the tests . . . You would get more tests delivered to the patients. While they’re [patients] here, you just fill out the paperwork, have them sign, send it back with the billing information, and then they mail it back to the company. It would work better, especially if there’s no fee for storing it or having it in office. (Focus group 1 [small, rural])

Abbreviations: CFIR, Consolidated Framework for Implementation Research; HPV, human papillomavirus.

a The CFIR (14) considers implementation-related factors in 5 major domains: 1) inner setting (potential facilitators and barriers that would affect willingness to implement a proposed intervention), 2) individual characteristics (perceived benefits to and potential areas of pushback against adopting a proposed intervention and appropriateness for patients’ needs), 3) outer setting (external pressures, performance metrics, or other considerations that would encourage or discourage efforts to improve a proposed intervention among patients), 4) intervention characteristics (resources needed to implement a proposed intervention), and 5) implementation process (how staffing, scope of practice, and workflows may need to change to allow implementation).

**Individual characteristics.** Participants consistently noted that offering HPV self-collection could increase access to and rates of screening among patients not routinely visiting the clinic. We found a high rate of acceptability of self-collection among FQHC staff. For in-clinic provision, most participants thought patients would try using the kit if given health care provider–delivered education about HPV and instructions on correct use of the kit. When comparing HPV self-collection to at-home fecal immunochemical tests (FITs) already in place for colorectal cancer screening, participants believed HPV self-collection would be feasible to implement.

**Intervention characteristics/implementation process.** Distribution of in-person, mailed, or community outreach kits was expected to have a positive effect on clinic quality and screening measures in the event that HPV self-collection is approved by FDA. Providing self-collection kits during community outreach events could reach a greater proportion of the population of underscreened patients, compared with in-clinic screening. Having an external company distribute kits, provide testing, and conduct billing could help defer clinic costs and the burden on billing departments.

**Inner setting.** Offering self-collection could make time available at clinic appointments for Papanicolaou (Pap) tests and save time for health care providers, allowing them to address other issues during a patient encounter.

Focus group participants also described concerns about self-collection for HPV testing, which were organized into these CFIR domains: individual characteristics/inner setting, intervention characteristics/implementation process, and outer setting (Table 2).

**Table 2:** Concerns About Implementing Self-Collection for HPV Testing in Federally Qualified Health Centers: Results of Focus Groups (N = 6) Conducted Among Clinical Personnel (N = 45), North Carolina, January 2020–March 2021

CFIR construct^a^	Theme	Illustrative quotes on concerns about HPV self-collection
Individual characteristics/inner setting	Acceptability	If they’re here, why not just do a Pap? I guess that’s . . . for us, we work in family medicine. So, it could be something that we give to them in addition. . . . But if they’re already gonna be on the OB [obstetrics] side, I don’t really see why they would need to do a self-collection for an OB appointment or even a gynecological appointment. (Focus group 2 [small, urban])
	Accuracy and reliability	• [I]s the accuracy as far as detecting the HPV as well as actual cervical swabs? . . . Or if it doesn’t adequately screen them, then time has already been wasted. (Focus group 2 [small, urban]) • I would be more concerned about getting false negatives. (Focus group 1 [small, rural]) • I would be kind of concerned about that because you get one chance . . . to get a good vaginal exam and testing. . . . [D]o I want to risk them doing it themselves and not getting a good sample? And then I’m having to convince them to either do it themselves again or come in and have it done. (Focus group 1 [small, rural]) • [S]helf life would need to be decent if it costs a lot [and is] a simple tube and swab. (Focus group 1 [small, rural])
	Reduction in direct patient–provider communication	[If] we’re distributing these kits . . . they still need to come to their providers. I think if we’re just distributing things, it just is kind of a way to keep them from coming in for issues that may need to be addressed in other ways. (Focus group 2 [small, urban])
	Financial impact: cost to center	• If we were to give them the kit while they were here . . . Do you bill it when you give it to them? Or do you bill it when you get it back? (Focus group 1 [small, rural]) • [If] we send them out, and don’t bill for them until they’re returned, I can see that being a big cost. . . . [A] lot of them may not be returned. So, we’re never gonna be able to bill the insurance for it. (Focus group 1 [small, rural]) • I think the payment structure and the support for it would be [one of] the two questions that have to be answered. (Focus group 5 [large, rural]) • That’s something that a lot of FQHCs [federally qualified health centers] struggle with because we don’t have funding for great EHRs [electronic health records]. And to get the necessary updates and everything, you have to pay all this money. And we just can’t do it. (Focus group 1 [small, rural])
Intervention characteristics/implementation process	Kit distribution in clinic	[I]f they’re in for something like a rash, and then you say . . . “Take this.”. . . [T]hat’s not going along with what you’re seeing them for, and it’s . . . part of an annual GYN [gynecologic] visit that you’re not even doing at that time. (Focus group 6 [medium, rural])
	Kit distribution via mail	I think we would just have to be really thoughtful about that process with the mail-out, just making sure that we’re hitting all avenues as far as explaining it to the patient, maybe doing some calls until it starts becoming more of a thing that people are like, “Oh, okay.” (Focus group 5 [large, rural])
	Community outreach events	• I don’t know if that’s . . . how you need to distribute this is through a community outreach type-thing. That might scare them away. (Focus group 2 [small, urban]) • At our community day . . . usually during National Health Center Week . . . only two people came. (Focus group 4 [medium, urban])
	Delivering test results	• [It’s] hard to track . . . incorrect phone numbers . . . mailing addresses . . . patients just not answering their phones or calling you back. (Focus group 1 [small, rural]) • [Y]ou would be surprised [by] the lack of knowledge people have. . . . I have to go through sometimes 10 to 15 min on the telephone explaining how your Pap may be normal, but your HPV is positive. (Focus group 2 [small, urban]) • [O]ur patients — if they have a patient portal — the moment any test results electronically back to the provider, it also automatically results back to the patient portal for that patient. . . . [T]hat’s good that they’re getting access to their results, but it’s also a big issue because now they can see results, and wonder what’s going on with those results before the provider even sees them – which can raise a big issue. (Focus group 3 [large, rural]) • We have a portal that nobody uses. . . . It’s not user friendly. (Focus group 1 [small, rural])
	Workflow	• [W]hat kind of support would we receive in implementing it? Because there’s not enough of us to go around as it is now. (Focus group 5 [large, rural]) • As long as the results aren’t anything weird. As long as you know what you’re reading . . . how to read it. (Focus group 2 [small, urban])
Outer setting	Quality measures	• [D]oes this even satisfy our quality measures? Is this even an approved test? (Focus group 6 [medium, rural]) • [Y]our biggest challenge is . . . does [this] … meet the HRSA [Health Resources and Services Administration] requirement for cervical cancer screening[?] . . . You get a little bit longer [between screening] if you have an HPV test, but you still have to have that cervical cytology to meet that guideline. . . . [U]ntil that changes, it’s sort of a hard sell. (Focus group 5 [large, rural])
	COVID-19	[W]e’ve essentially prioritized COVID vaccinations. . . . For something that doesn’t meet a UDS [Uniform Data System] measure . . . to dedicate staff time to it in the COVID season is just probably not gonna happen. (Focus group 5 [large, rural])

Abbreviations: CFIR, Consolidated Framework for Implementation Research; HPV, human papillomavirus.

a The CFIR (14) considers implementation-related factors in 5 major domains: 1) inner setting (potential facilitators and barriers that would affect willingness to implement a proposed intervention), 2) individual characteristics (perceived benefits to and potential areas of pushback against adopting a proposed intervention and appropriateness for patients’ needs), 3) outer setting (external pressures, performance metrics, or other considerations that would encourage or discourage efforts to improve a proposed intervention among patients), 4) intervention characteristics (resources needed to implement a proposed intervention), and 5) implementation process (how staffing, scope of practice, and workflows may need to change to allow implementation).

**Individual characteristics/inner setting.** Participants felt a risk to self-collection was being repetitive of in-clinic gynecologic examinations or replacing Pap tests. The accuracy and reliability of self-collection kits were questioned: the potential for user and test error could lead to inaccurate, “useless,” and false-negative results, which could deter patients from follow-up testing. For inaccurately collected samples, health care providers would have to persuade patients to reattempt self-collection or return for an in-clinic Pap–HPV co-test. With inadequate HPV test results, additional time would be required to have patients rescreened, thus delaying the screening process. Concerns were raised about the shelf life of self-collection kits housed in clinics. The use of kits distributed outside the clinic could reduce the opportunity for direct patient–provider communication and the opportunity for patients to be seen directly for other health issues. Participants requested clarification about financial aspects (eg, insurance billing, payment structure) and the potential burden of cost on the FQHC.

**Intervention characteristics/implementation process.** Participants expected difficulties with distributing kits via mail such as having inaccurate patient contact information, needing to be thoughtful about patient reactions upon receiving the kits without advance notice, and having to call patients to provide sufficient education and guidance on self-collection to ensure collection accuracy. Participants discussed previous attempts at offering FITs through a community outreach initiative in-clinic and at community health fairs, although they noted the lack of patient attendance. Relaying self-collection results to patients was considered challenging because of unreliable patient contact information, difficulty navigating patient portals, and low health literacy among patients. Workflow disruptions might arise, given the need for staff training on how to interpret self-collection results and for various resources needed to support implementation.

**Outer setting**. Given that HPV self-collection is not yet FDA-approved, a related concern was whether self-collection would satisfy Health Resources and Services Administration (HRSA) requirements for cervical cancer screening. If self-collection does not meet these health care guidelines, it would pose a problem with underreporting for FQHCs, which are held accountable for achieving specific performance measures. Furthermore, several health care providers at the time of interview indicated that current priorities had shifted to focus on the COVID-19 pandemic, making it difficult to implement HPV self-collection.

### Semistructured interviews

Interview participants described several benefits of self-collection for HPV testing, which were organized into 4 CFIR domains: individual characteristics/inner setting, implementation process/inner setting, intervention characteristics/implementation process, and inner setting (Table 3).

**Table 3:** Benefits of Implementing Self-Collection for HPV Testing in Federally Qualified Health Centers: Results of Key Informant Interviews Conducted Among Chief Executive Officers, Senior-Level Administrators, and Chief Medical Officers (N = 18), North Carolina, May–September 2021

CFIR construct^a^ and theme	Illustrative quotes on benefits of HPV self-collection		
	Chief executive officers	Senior-level administrators	Chief medical officers
**Individual characteristics/inner setting**			
Increase screening rates and care access	• I’d be very interested . . . in anything that can be done that would help us get the community healthier or . . . identify problems. (ID 005) • I think being as creative and innovative as possible to make sure . . . how can we be unique and inviting self-collecting screenings for our patients. (ID 010)	• [Y]ou’d have a really good promise of increasing those testing rates if it’s something that's simple . . . [and] not costly. (ID 024) • [L]ife is so hard on a lot of women because they have a hundred and one things that they’re doing. So, having something like this where they can simply do it at home or do a quick bathroom test, and some people are just terrified to come and do that. . . . Sometimes there is no woman in the clinic for months . . . [T]hat particular patient doesn’t feel comfortable going to the male physician. (ID 012)	• [I]t would definitely help our cervical screening rates. (ID 013) • [F]or the target population that we would miss at those annual visits, that are not gonna come in regardless, there may be more an opportunity for them to follow up hopefully with those self-collection and need to come in, and are understanding kind of the urgency. (ID 022) • [It] gives women more options to do cervical cancer screening, and . . . result in a higher percentage of women who are due for it, getting it. (ID 001)
Acceptability	—	[I]f the tests were shown to be reliable and accurate and easy for patient use and cost-effective for both the center and the patient . . . [providers] would be on board with getting it done and wanting . . . it here and using it . . . a lot. (ID 008)	[For] support inside, of course, you definitely have the CMO [chief medical officer] and our clinical director on board and provider staff and . . . the entire clinical department which involves providers and nurses. (ID 013)
Identify issues earlier	[R]educe the risk of developing cancer or catching it early enough to prevent it or treat it. (ID 010)	—	[W]e’d be able to pick up on the HPV. . . if we let the patient take it home as long as they would return it. (ID 006)
Promote patient inclusion in their own health and well-being	—	[O]pportunity to engage the patient so they see that they are an active participant in their own health. (ID 019)	• [P]eople can have a choice on what they feel like is the best [screening] option for them. (ID 001) • [O]pportunity to have a conversation for the need for a more invasive test. And . . . a more shared decision about continuing on with the next step. (ID 022)
**Implementation process/inner setting**			
Feasibility/workflow	• Probably not a lot [of change needed]. . . . [Y]ou could probably work that in with regular workflow. (ID 023) • [T]he physicians that work in federally qualified health centers . . . are about educating their patients about that annual Pap smear [Papanicolaou test]. . . . I can see them easily incorporating [self-collection] into their discussion with their patients. (ID 023)	[W]e have the staffing to where we wouldn’t need to add additional staff. (ID 008)	• [W]e’ve been doing more telehealth. . . . So, we're already sending patients home with FIT [fecal immunochemical test) kits to do in their home. . . . [T]hat initiation was really quite easy. And I can see the HPV self-collection being just as easy and maybe even easier. (ID 009) • I don’t actually think it would be very hard at all to start doing this in our clinics. (ID 001)
Able to operate at a support-level staff	[Y]ou might even have your CMA [certified medical assistant] help with that, so they could be trained to instruct more, and it doesn’t have to be a provider. (ID 023)	A lot of times, we can have the nurses help. . . . [I]f the nurse was the one seeing the patient, we can see a lot of women during the day. (ID 012)	[T]hat can be done on more of a support staff level. It doesn’t necessarily have to really involve the physician if we have standing orders. Unlike . . . cytology where I have to do the procedure. (ID 009)
**Intervention characteristics/implementation process**			
Kit distribution in clinic	• [I]f it’s in clinic, [it’s] less of an administrative burden. (ID 003) • [S]end them to the bathroom or they can do it right there in the exam room as the provider leaves and then get it from them after they’re done. . . . I don’t . . . perceive a whole lot of barriers to it all. (ID 003) • The provider and the nursing staff could educate the patient on how to do a self-collection test, . . . show them a diagram of the anatomy, and explain how to do the collection on that diagram. [T]hat patient would be right there in the office and can maybe even have a staff person present in the room while the patient self-collects and if having any problems or questions, the staff person can be right there in the room to help. (ID 014)	When the provider actually hands them one in the office and says, “You don’t want to get your Pap smear but this is a good other option,” and they walk them through it, we find we get the best results that way. So, if it’s actually sitting here in my office that I can actually hand a patient, that’s the best way to get it to them. . . . And they’ll go ahead and potentially just do it and deal with it . . . . [W]hen a provider talks to them, right then, there’s more of an urgency they feel. (ID 004)	[O]ur goal is really to look at the quality and screening for every visit. And, so, any visit where they’re there, I can say, “[I]t looks like you’re due . . . for your cervical cancer screening, so why don’t I send this home with you, and this is what you do. You can mail it back, and we’ll let you know the results.”. . . [B]ut, in general, I also give them an opportunity to do the Pap smear with us if they prefer that. . . . [T]hey can have a choice. (ID 001)
**Inner setting**			
Financial impact: funding	—	[I]f we internally took this on as an improvement issue, as we are concerned about our cervical cancer screening rates, and we set it as a goal to improve, then we would dedicate resources to it . . . use our revenue . . . our profit. (ID 015)	—

Abbreviations: —, no relevant quotes; CFIR, Consolidated Framework for Implementation Research; HPV, human papillomavirus.

a The CFIR (14) considers implementation-related factors in 5 major domains: 1) inner setting (potential facilitators and barriers that would affect willingness to implement a proposed intervention), 2) individual characteristics (perceived benefits to and potential areas of pushback against adopting a proposed intervention and appropriateness for patients’ needs), 3) outer setting (external pressures, performance metrics, or other considerations that would encourage or discourage efforts to improve a proposed intervention among patients), 4) intervention characteristics (resources needed to implement a proposed intervention), and 5) implementation process (how staffing, scope of practice, and workflows may need to change to allow implementation).

**Individual characteristics/inner setting.** CEOs, senior-level administrators, and CMOs were highly receptive to adopting self-collection kits at their FQHCs, mentioning it could increase screening rates and access to care. Senior-level administrators were receptive if kits were reliable, accurate, easy to use, and cost-effective. Self-collection was considered an opportunity to increase access to care among patients who feel apprehensive about being screened by a male physician. As with in-clinic testing, self-collection can reduce the risk of cervical cancer through early HPV detection. Self-collection may foster an opportunity to encourage patient engagement in and ownership of their own health and patient–provider shared decision-making about care.

**Implementation process/inner setting.** After HPV self-collection processes were described, interviewees from the 3 roles felt that existing staff could support self-collection processes, rather than needing to hire additional staff. Referring to prior experience with implementing FITs, CMOs believed HPV self-collection kits might be easier to implement.

**Intervention characteristics/implementation process.** CEOs believed that distributing HPV self-collection kits in clinics would be less of an administrative burden than mailing kits to patients, which would involve identifying patients and processing returned kits. Furthermore, with in-clinic distribution, a staff member could demonstrate the collection procedure by using illustrations and diagrams and be available to assist if requested. Senior-level administrators and CMOs felt patients might be more likely to return the kits if they received them directly from their health care provider rather than via mail. Interviewees envisioned that if health care providers distributed kits, they could then emphasize the importance of screening to instill a sense of urgency and describe screening options to provide patients with a choice.

**Inner setting.** Senior-level administrators noted that if self-collection were HRSA approved and presented as a method to improve cervical cancer screening at their clinical sites, FQHCs could more feasibly generate revenue and profits and dedicate the needed resources toward implementation.

Interview participants also described concerns about self-collection for HPV testing, which were organized into the following CFIR domains: individual characteristics/inner setting and intervention characteristics/implementation process (Table 4).

**Table 4:** Concerns About Implementing Self-Collection for HPV Testing in Federally Qualified Health Centers: Results of Key Informant Interviews Conducted Among Chief Executive Officers, Senior-Level Administrators, and Chief Medical Officers (N = 18), North Carolina, May–September 2021

CFIR construct^a^ and theme	Illustrative quotes on concerns about HPV self-collection		
	Chief executive officers	Senior-level administrators	Chief medical officers
**Individual characteristics/inner setting**			
Acceptability	[I]f the FDA [US Food and Drug Administration] would approve some collection, I would hope the government would add that self-collection as an acceptable method within cervical screening measure and then the other insurance companies would also follow suit and accept it as well. (ID 014)	—	• [I]t would just really depend a lot on giving providers the proper information to educate their patients . . . making sure that we knew as much about the collection process, and the reliability of the test. (ID 001) • Not today . . . there would need to be . . . more of a structure and understanding of the role for it . . . more . . . education on how it works . . . the sensitivities and specificities of the test, and how it would fit in with some of the . . . standard of care. (ID 022)
Are kits complementary or competitive? Papanicolaou test (Pap smear) replacement	• [P]robably more in competition. . . .[W]hat’s the point of doing a self-collection if you’re doing a Pap anyway? . . . [Y]ou can easily assess for HPV with the Pap [co-testing] as well. . . . [I]t would be just a waste to do both. (ID 014) • [Replacing the Pap smear is] . . . what you don’t want to happen because there’s so much there that needs to be seen and . . . evaluated. (ID 023)	[C]omplementary if administered correctly . . . if you’re dealing with a patient who doesn’t want to even engage in sexual health and women’s health care. That’s barrier number one, and then you’re gonna add a more complex component of . . . a self-swab, and the patient doesn’t even want to have the exam, period. . . . [Y]ou can’t go from nothing to “now I want you to engage and do it yourself” overnight. (ID 019)	• A little bit in competition with one another. . . . Our Pap smears, now they automatically do HPV. . . . [M]aybe if you had a patient who didn’t want to do a Pap smear but would do a self-collection. (ID 006) • [T]here may be a misconception of, “Well, this is all I have to do. I don’t necessarily need to follow up with [a Pap smear or] anything else.” (ID 022)
Accuracy and reliability	[I]f their HPV was positive on self-collection, I’d say they’d need to undergo a dedicated pelvic exam by a health care provider and then also undergo a formal Pap smear as well . . . either with the PCP [primary care provider] . . . or GYN [gynecologist]. (ID 014)	Yes [patient needs to be rescreened in clinic after positive HPV self-collection result] . . . whatever pertinent processes that are the best practices for positivity should ensue. (ID 019)	•[I]f they’re positive, they need a follow up — a Pap smear and a self-collection. (ID 022) • [I]f somebody self-collects and their HPV’s negative, that doesn’t necessarily mean that those cells at the cervix would be normal. (ID 022) • [Y]es [patient needs to be rescreened after positive HPV self-collection result]. . . . [I]t depends on their history. . . . [H]ave they had a history of dysplasia? HPV in the past that’s been consistent? (ID 018) • Is that self-collection picking up higher-risk HPV or . . . just any type of HPV? (ID 022) • [Patients who have positive test results from HPV self-collection kit] should need to be [rescreened] . . . just to make sure that those results were correct. (ID 006) • [A]ssuming our specificity is good . . . we would move forward with next steps . . . as long as the test itself was statistically good, then I don’t think we’d repeat it. (ID 009)
Reduction in direct patient–provider communication	—	—	[M]ake sure that folks were getting the rest of their preventative care too. Some of which will only be done if they’re in the office. (ID 009)
Financial impact			
Billing/cost	• [We] have to look at the expense of the kits, how much it actually cost [and] factor that into how do we generate revenue to cover that? (ID 010) • If we had to pay for those kits . . . [it would] reduce our ability to be able to be a participant. . . . [O]ur payer mix is very one-sided as far as Medicaid and self-pay patients. (ID 005) • [W]ho’s paying for the test? . . . . How much does this test cost? If it’s a hundred dollars . . . that’s gonna be a problem if nobody’s paying for it. We could end up trying to get grants for it and do the same things that we do as a community health center to get these things into the hands of patients that can’t afford them. (ID 003)	[O]nce it’s approved, is that screening tool that BCCCP [Breast and Cervical Cancer Control Program] would pay for? You know, or is there a grant that pays for this for so long until data can be figured out? (ID 004)	[B]udget-wise, our patients don’t pay anything out of pocket for their laboratories outside of their copay. . . . [W]e need just to think about from a budget perspective, how this looks versus our current cytology plus HPV and . . . if we needed to adjust that. (ID 009)
Number of patients seen in a day	[I]f you [are] looking at [a] decrease [of] one, two, three, four, five patients a day, you [are] looking at close to an average of two hundred dollars, two hundred and fifty dollars per patient that comes through . . . [T]he price of the kit might be . . . cost-prohibitive for us . . . [F]inancially, we just have to be careful because we operate pretty close to our budget. (ID 005)	—	—
**Intervention characteristics/implementation process**			
Kit distribution via mail	[I]f we had to mail these out and do it . . . that will add an administrative burden to it. . . . If we had to send it out, find out who they are, mail it to them. You know, sometimes the address is incorrect. (ID 003)	—	—
Delivering test results	—	—	[P]hone numbers change like the wind here . . . so the big barrier is getting ahold of patients to get that documented back to see what’s going on . . . even addresses have changed. (ID 006)
Workflow	• [F]rom the health center standpoint . . . [we] would have to do some training with the staff about it. In terms of billing and coding and that kind of thing. (ID 014) • [There would need to be] administrative training around the EHR [electronic health record]. (ID 010)	• [O]n behalf of community health centers . . . we are overwhelmed with staff who have to wear multiple hats. (ID 015) • [T]he clinical workflow will become more expansive and again addressing health literacy, hesitancy, education, and ensuring equity in approach. (ID 019)	• Right this very moment . . . staffing shortages . . . that might be worth a conversation to revisit. (ID 018) • Staff, cultural work would have to be done with staff and providers. (ID 018) • [Train staff] specifically just for the self-collection. (ID 022) • [S]taffing would just be a real issue as far as the tracking and things of that nature. (ID 013)

Abbreviations: —, no relevant quote; CFIR, Consolidated Framework for Implementation Research; HPV, human papillomavirus.

a The CFIR (14) considers implementation-related factors in 5 major domains: 1) inner setting (potential facilitators and barriers that would affect willingness to implement a proposed intervention), 2) individual characteristics (perceived benefits to and potential areas of pushback against adopting a proposed intervention and appropriateness for patients’ needs), 3) outer setting (external pressures, performance metrics, or other considerations that would encourage or discourage efforts to improve a proposed intervention among patients), 4) intervention characteristics (resources needed to implement a proposed intervention), and 5) implementation process (how staffing, scope of practice, and workflows may need to change to allow implementation).

**Individual characteristics/inner setting.** Several CEOs were hesitant about implementing HPV self-collection because of the lack of FDA approval. CMOs preferred to delay implementing HPV self-collection because of several uncertainties, such as the reliability of self-collection and the time available for health care providers to educate patients about the self-collection process and integrate it into the standard of care. CEOs and CMOs believed an HPV self-collection strategy could compete with current in-clinic cervical cancer screening methods, and they often expressed concern about the HPV test replacing the Pap test. Some senior-level administrators questioned whether self-collection was appropriate and acceptable for their FQHC patient population, who might be uncomfortable actively engaging in discussions about sexual and women’s health. Interviewees lacked consensus as to whether a follow-up to rescreen with cytology was necessary after a positive self-collection test result. One CMO felt that if patients received negative HPV self-collection test results, they could still have abnormal cytology. Other CMOs expressed concern that implementing HPV self-collection could reduce the frequency of patients seeking in-clinic preventive care. Interviewees wondered about the financial aspect of paying for the kits and reimbursing costs to health care providers. CEOs inquired about the cost of kits, how to generate revenue, and the financial impact if implementation resulted in decreases in patient visits. CMOs discussed the need to understand how current billing criteria would need to be adapted for self-collection versus an in-clinic routine Pap–HPV co-testing examination, since most FQHC patients generally do not pay more than their copayment.

**Intervention characteristics/implementation process. **CEOs believed that distribution of self-collection kits via mail would add an administrative burden because of the difficulty in identifying patients due for screening, correcting inaccurate contact information, following up with patients to return their kit, and communicating self-collection test results to patients. CEOs, senior-level administrators, and CMOs believed that introducing self-collection could potentially cause a disruption in workflow because of the need for training on education, billing, coding, and EHRs, and the possibility of staff shortages.

## Discussion

This study of FQHCs is among the first to examine the perspectives of clinical personnel on health service delivery considerations for implementing self-collection for HPV testing into clinical care. FQHC leadership and clinical staff found HPV self-collection to have clear potential to increase access to and rates of cervical cancer screening and be feasible to implement. HPV self-collection was considered an acceptable alternative to in-clinic co-testing if results were proven to be equally accurate for detection of cervical precancer/cancer, patients were provided proper education on self-collection, and the self-collection process was appropriately adapted to clinic workflow. Before implementation, financial uncertainties and the effect on quality measures would need to be addressed.

We used CFIR to identify considerations needed for a successful intervention. Providing patients with a screening option to complete HPV self-collection either in clinic or at home could reach a population of patients who would have missed their recommended screening visit and could subsequently result in higher rates of screening completion. When compared with providing a FIT kit in clinic for use at home, HPV self-collection implementation was considered highly feasible. Health care providers indicated that FQHCs could model FIT implementation, while addressing the logistical considerations specific to HPV self-collection.

Implementing HPV self-collection was considered an acceptable alternative to in-clinic co-testing by clinic staff and leadership once specific prerequisites are addressed. Self-collection screening must produce accurate results (ie, high sensitivity and specificity to detect high-grade precancer) that compare favorably with standard provider collection. Patients and clinical staff would need proper education on the self-collection process. Given that FQHCs emphasize patient education, study participants believed it would be a straightforward process to incorporate counseling discussions on self-collection. Implementation of HPV self-collection was considered a potential timesaver because health care providers would not need to conduct the initial screening procedure during an in-clinic pelvic examination; they would only need to see patients with a positive self-collection test result. However, implementation of self-collection might shorten the patient–provider encounter time in which health care providers often counsel their patients while conducting screening procedures (18). Additionally, FQHCs could establish workflow procedures to correctly identify patients due or overdue for screening and distribute self-collection kits in the clinic, via mail, or during community outreach — or in any combination of these 3 delivery methods. 

Clinic administrators wanted to consider the financial impact of HPV self-collection to understand insurance coverage, billing practices, clinic reimbursements, and payment structures that accompany implementation. This clarification was perceived as necessary to understand the financial impact of offering self-collection testing on clinic productivity and financial well-being, specifically, a decrease in patient visits and the possibility that HPV self-collection does not become an FDA-approved screening method.

Our findings add evidence to the literature on HPV self-collection acceptability among clinical personnel. While most studies of HPV self-collection assessed general patient and provider acceptability and test accuracy, our qualitative findings are unique in that they focus on specific health service delivery considerations for implementing HPV self-collection. These include financial impact, workflow implications, and methods for kit distribution. In a Canadian study, Muslim women reported that unless costs of HPV self-collection were covered by a public health program or health insurance, they would not participate in a self-collection screening program (19). Similarly, our study participants expressed hesitancy with proceeding with HPV self-collection implementation because of the financial uncertainties, including when to bill for kits, funding for implementation, payment structure, cost of kits, and financial implications of a potential reduction in patient volume. Despite cost concerns, our previous My Body, My Test 3 study found that of 227 low-income women in North Carolina who returned self-collection kits and completed an acceptability questionnaire, 92% were willing to pay for the kits themselves, with 48% willing to pay $25 or more (12). However, staff support will also be needed to accommodate the implementation for billing, maintaining EHRs, staff training, and offering testing-related education and assistance to patients (eg, responding to questions).

Consistent with previous studies in the US (19–21), our clinical personnel raised concerns about their patient’s ability to collect adequate samples, the performance of the self-collection test, and patient adherence to provider recommendations. A lack of patient adherence would lead to missed preventive examinations and clinic-based follow-up procedures, further leading to missed opportunities to address other health-related issues (19,21). Despite these concerns, many domestic and international health care providers have found HPV self-collection to be highly acceptable before and after trying self-collection (19–26). In one study, 80% of clinical staff surveyed in 2 safety-net clinics in Florida were willing to incorporate self-collection into practice (22). Among Australian health care providers, 64% of general practitioners, obstetricians, and gynecologists found self-collection to be a reasonable alternative to practitioner-collected HPV screening; the remaining health care providers believed targeted education would best address underscreened women (25).

Our findings echo the need for education on the self-collection process among health care providers and patients (19,21). Health care workers are strong proponents of patient-centered education on self-collection because it can lead to increased uptake of self-collection for HPV testing, better sample collection, and improved confidence and willingness of patients to self-collect (19). Although our study participants expressed concerns that implementing self-collection might reduce patient–provider interaction, not all health care workers agreed (19). A meta-analysis found that several physicians and nurses saw the value in women performing self-collection at home and argued it would be beneficial if patients came to the health center to receive their results, because this would also serve as an opportunity for further discussion and clinical examination if needed (19). Clinical collaborators expressed some hesitation about mailing kits to patients because of cost, safety, privacy, and identifying eligibility (24). Interestingly, an Australian study found that use of a self-collection pathway was driven more opportunistically by health care providers in clinic than by patients themselves (24). Studies in Canada and Kenya emphasized the importance of political engagement and support for successful operationalization and implementation of an HPV self-collection program, which was not touched on in our discussions (19).

### Strengths and limitations

Strengths of our study include the focus on FQHCs and the unique and diverse patient population they serve. Our approach used focus groups and in-depth interviews for a personal and interactive engagement to elicit insightful results on facilitators and barriers to implementing HPV self-collection and health care delivery considerations for implementation. Our study also had several limitations. Participants were selected from 6 of the 40 eligible FQHCs in North Carolina. This selection of FQHCs may affect the generalizability of populations served by other FQHCs. Given that the study was conducted during the COVID-19 pandemic, in-person interviews were not possible. Virtual meetings might have resulted in less personal connections than in-person meetings would have. Participants also received monetary compensation for participating, which may have had an unidentified effect on their motivation and attitudes during the study. Furthermore, we did not assess acceptance of HPV self-collection among the FQHCs’ patient population. Although our study participants reported hesitancy about patient acceptance, the literature states otherwise (9–13). The opportunistic distribution of HPV self-collection kits in FQHCs would still not address the problem of patients overdue for screening and not attending their clinic visits.

Our findings have implications not only for implementing HPV self-collection but for other novel methods for screening. Self-collection has been studied, developed, and implemented for other sexually transmitted infections, colorectal cancer screening, HIV, and COVID-19 (27,28). Priorities for future research include evaluation of the effectiveness of implementing HPV self-collection on cervical cancer screening rates with real-world implementation, access to care, and any related social harms; unintended consequences upon receipt of other novel and evidence-based screening services for public health interventions; the impact of implementing HPV self-collection on quality performance measures; and health service delivery considerations for implementing other novel screening technologies in both international and domestic settings.

#### Conclusion

Implementing self-collection for HPV testing at FQHCs in North Carolina was found to be highly acceptable and feasible among FQHC leadership and frontline clinical staff. Our findings contribute to promising evidence that HPV self-collection offers an alternative approach to improving cervical cancer screening coverage and provides important considerations for its successful implementation. Despite the positive outlook on the implementation of HPV self-collection, multiple challenges must be addressed before incorporating HPV self-collection into the standard of care.

## Post-Test Information

To obtain credit, you should first read the journal article. After reading the article, you should be able to answer the following, related, multiple-choice questions. To complete the questions (with a minimum 75% passing score) and earn continuing medical education (CME) credit, please go to http://www.medscape.org/journal/pcd. Credit cannot be obtained for tests completed on paper, although you may use the worksheet below to keep a record of your answers.

You must be a registered user on http://www.medscape.org. If you are not registered on http://www.medscape.org, please click on the “Register” link on the right hand side of the website.

Only one answer is correct for each question. Once you successfully answer all post-test questions, you will be able to view and/or print your certificate. For questions regarding this activity, contact the accredited provider, CME@medscape.net. For technical assistance, contact CME@medscape.net. American Medical Association’s Physician’s Recognition Award (AMA PRA) credits are accepted in the US as evidence of participation in CME activities. For further information on this award, please go to https://www.ama-assn.org. The AMA has determined that physicians not licensed in the US who participate in this CME activity are eligible for AMA PRA Category 1 Credits™. Through agreements that the AMA has made with agencies in some countries, AMA PRA credit may be acceptable as evidence of participation in CME activities. If you are not licensed in the US, please complete the questions online, print the AMA PRA CME credit certificate, and present it to your national medical association for review.

## Post-Test Questions

### Study Title: Self-Collection for Primary HPV Testing: Perspectives on Implementation From Federally Qualified Health Centers

#### CME Questions

1. Which one of the following statements regarding self-collected samples for cervical cancer testing is most accurate?
  - The US Preventive Services Task Force (USPSTF) recommends self-collected samples for human papillomavirus (HPV) testing among women at age 30 years and older
  - HPV self-collection lacks sensitivity for high-grade cervical lesions compared with provider collection
  - HPV self-collection has not been demonstrated to improve screening uptake
  - The majority of providers in previous research have been willing to adopt HPV self-collection
2. Which one of the following benefits of HPV self-collection was most salient among participants in the current study?
  - Self-collection could provide extra revenue for the clinic
  - Self-collection would be empowering for patients
  - Self-collection could increase the screening rate for cervical cancer
  - Self-collection would reduce staff training time
3. Which one of the following was a common concern regarding self-collection in the current study?
  - Self-collection may be inaccurate, especially as a result of user error
  - Self-collection would result in more cervical cytology studies
  - Self-collection could result in patient injury
  - Self-collection could lose US Food and Drug Administration (FDA) approval with widespread use
4. Which one of the following methods for distribution of HPV self-collection kits was most favored by federally qualified health center leadership in the current study?
  - Direct provision of kits to patients in the clinic
  - Mailing kits to patients’ homes
  - Making the kits available at large community-based health fairs
  - Contracting with a third party to distribute kits and manage results
